# Research on the Postural Stability of Underwater Bottom Platforms with Different Burial Depths

**DOI:** 10.3390/s24103034

**Published:** 2024-05-10

**Authors:** Yong Wei, Nan Li, Ming Wu, Daming Zhou

**Affiliations:** 1 Naval Submarine Academy, Qingdao 266199, China; 2 No. 710 Research Institute of China Shipbuilding Industry Corporation, Yichang 443003, China; 3 School of Astronautics, Northwestern Polytechnical University, Xi’an 710072, China

**Keywords:** bottom platform, underwater sensors, computational fluid dynamics, burial depths, postural stability

## Abstract

The bottom platform is an important underwater sensor that can be used in communications, early warning, monitoring, and other fields. It may be affected by earthquakes, winds, waves, and other loads in the working environment, causing changes in posture and affecting its sensing function. Therefore, it is of practical engineering significance to analyze the force conditions and posture changes in the bottom platform. In order to solve the problem of postural stability of the underwater bottom platform, this paper establishes a fluid and structural simulation model of the underwater bottom platform. First, computational fluid dynamics (CFD) technology is used to solve the velocity distribution and forces in the watershed around the bottom platform under a 3 kn ocean current, where the finite element method (FEM) numerical calculation method is used to solve the initial equilibrium state of the bottom platform after it is buried. On this basis, this paper calculates the forces on the bottom platform and the posture of the bottom platform at different burial depths under the action of ocean currents. Additionally, the effects of different burial depths on the maximum displacement, deflection angle, and postural stability of the bottom platform are studied. The calculation results show that when the burial depth is greater than 0.6 m, and the deflection angle of the bottom platform under the action of the 3 kn sea current is less than 5°, the bottom platform can maintain a stable posture. This paper could be used to characterize the postural stability of underwater bottom platforms at different burial depths for the application of underwater sensors in ocean engineering.

## 1. Introduction

The bottom platform is an important underwater sensor that can be applied in communications, early warning, monitoring, and other fields. In its working environment, it may be subjected to loads such as earthquakes, winds, and waves, which can cause changes in posture and affect its functionality. Therefore, analyzing the stress conditions and posture changes in the bottom platform has practical engineering significance for underwater sensors.

Various methods have been used to study the interaction of this pile–soil structure. The literature [1] presents a review of the seismic design and evaluation of diverse structures, highlighting the significance of integrating soil–structure interaction with depth-varying spatial effects. The literature [2] investigates the cyclic response mechanism of offshore wind turbines supported by piled jackets through the utilization of an aero-elastic model, enhanced with diverse pile–soil interaction models. In the literature [3], the vertical vibration characteristics of an offshore end-bearing pile embedded in saturated soils are investigated using a semi-analytical approach. The modeling outcomes reveal that factors such as water depth, pile dimensions, and soil properties exert significant influence on the response of offshore pile vertical vibration. In the literature [4], an analytical framework is formulated to assess the dynamic impedance of pile groups comprising an arbitrary number of cylindrical piles interconnected by a rigid cap. The solution takes into account the interplay between the secondary ground wave effects transmitted via pile–soil vibration and the hydrodynamic pressure arising from the pile–water interaction. The literature [5] first employs electrolytic corrosion experiments to produce steel samples with varying degrees of corrosion. Subsequently, it examines the impact of corrosion on soil–steel interfacial shearing properties through interfacial shearing tests and discrete element simulations. The literature [6] uses the finite element method to construct a marine platform pile leg model, and three finite element modeling methods simulate the interaction between the pile and the soil. Through a comparison of calculation results with actual data, they analyzed the accuracy and feasibility of the three simplified methods.

Previous studies generally considered the interactions between piles and soil but not the role of ocean currents. This study focuses on a cylindrical underwater bottom platform as the subject of investigation. Utilizing computational fluid dynamics (CFD) techniques and the finite element method (FEM) for one-way fluid–structure interaction analysis, the influence of burial depth on the postural stability of the underwater bottom platform was studied. The appropriate burial depth under the effect of ocean currents was determined. Furthermore, the stress and deformation of the soil around the platform were studied. This paper could provide valuable technical insights for the engineering applications of underwater bottom platforms.

The remaining sections of this paper are as follows: Section 2 introduces the simulation modeling details, including the geometric models, the fluid simulation model, and the structural simulation model. Then, in Section 3, the analysis of simulation results is presented. The conclusion is presented in Section 4.

## 2. Simulation Modeling

### 2.1. Geometric Models

**Figure 1:**
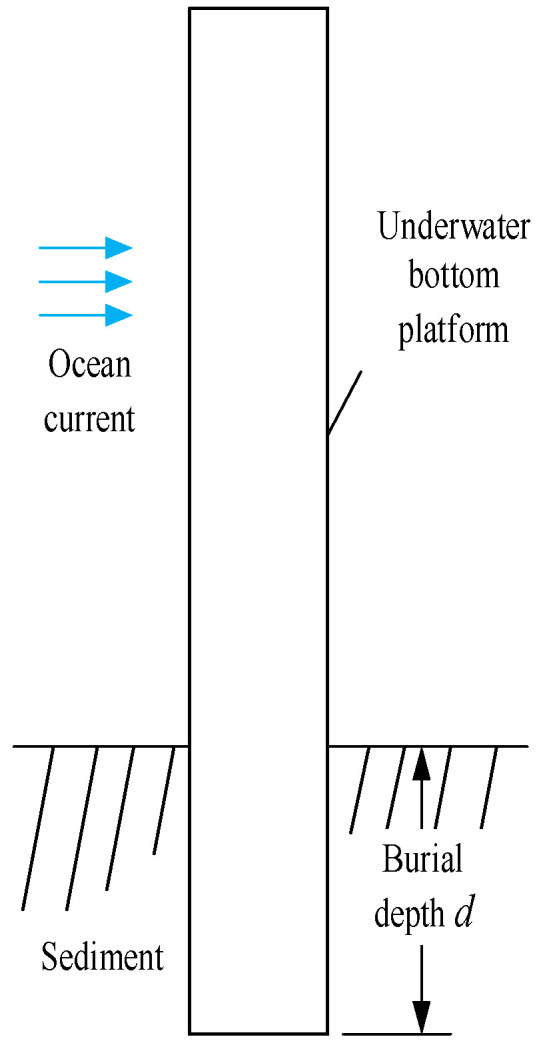
The structure of the proposed underwater bottom platform.

The underwater bottom platform utilizes its own negative buoyancy and high sinking speed during deployment to directly penetrate the seabed or adopts a self-burying device to achieve a partially buried state. This improves the platform’s postural stability under the influence of ocean currents. The structure of the proposed underwater bottom platform is shown in Figure 1.

### 2.2. Model Assumptions

The simulation should be based on the following assumptions:

(1) The influence of the platform on the surrounding flow field is neglected, and only the effect of the ocean currents on the platform is considered. Therefore, the fluid forces acting on the platform are assumed to be constant.
(2) The platform structure has high stiffness, and any structural deformations caused by the flow field can be ignored. Therefore, the platform can be treated as a rigid body.

### 2.3. Fluid Simulation Model

#### 2.3.1. Control Equations

Considering the platform operational conditions, the flow around the cylinder discussed in this paper involves high Reynolds number turbulence. To simulate this, the realizable turbulence model is applied, which is suitable for complex shear flows with slight rotation, vortices, and localized excessive flow. Its equation is provided as follows [7]:

(1) $$\frac{\partial }{\partial t}\left(\rho \varepsilon \right)+\frac{\partial }{\partial x_{i}}\left(\rho \varepsilon u_{i}\right)=\frac{\partial }{\partial x_{i}}\left[\left(\mu +\frac{\mu_{1}}{\sigma_{\varepsilon }}\right)\frac{\partial \varepsilon }{\partial x_{j}}\right]+\rho C_{1}S\varepsilon -\rho C_{2}\frac{\varepsilon^{2}}{k+\sqrt{v\varepsilon }}+C_{1\kappa }\frac{\varepsilon }{k}C_{3\varepsilon }G_{h}+S_{e}$$

where $C_{1}=\max\left[0.43,\frac{\eta }{\eta +5}\right]$; $\mu_{1}$ is the turbulent viscosity coefficient related to the strain rate, where $\mu_{1}=\rho C_{\mu }\frac{k^{2}}{\varepsilon }$; and k and ε are the turbulent kinetic energy and dissipation terms, respectively.

#### 2.3.2. Mesh Model

**Figure 2:**
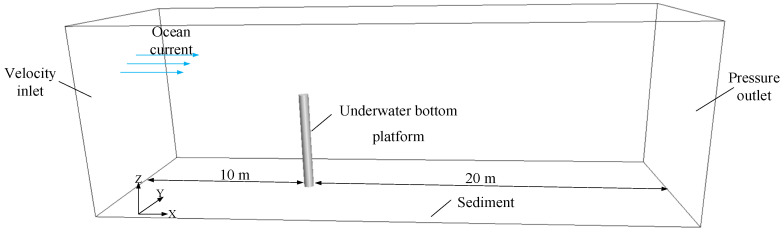
Schematic diagram of the fluid simulation calculation domain.

A cuboid computational domain is established outside the underwater bottom platform, with a size of 30 m × 10 m × 10 m, as shown in Figure 2. The front end of the computational domain is 10 m away from the platform, and the back end is 20 m away.

**Figure 3:**
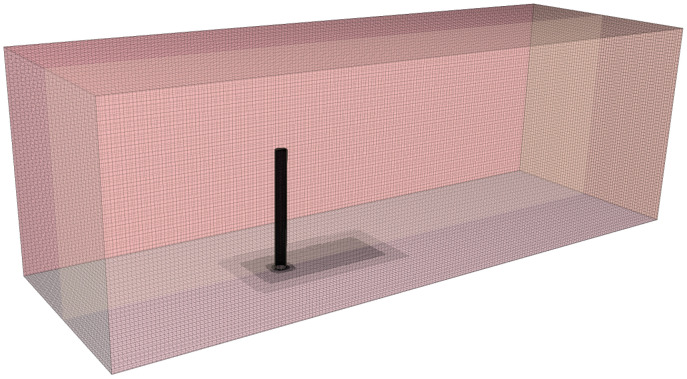
Schematic diagram of the mesh of the fluid simulation model.

**Figure 4:**
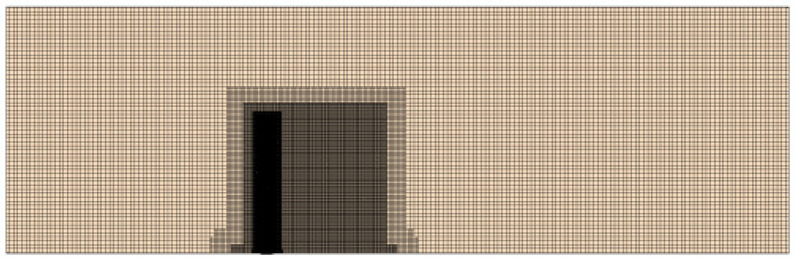
Schematic diagram of the mesh refinement of the fluid simulation model.

As depicted in Figure 2, a high-quality hexahedral mesh is generated to accurately simulate the boundary layer transition phenomenon of the cylindrical flow and the flow characteristics near the wall region of the wake of the cylinder. A prismatic layer grid is generated on the surface of the underwater bottom platform, and the mesh in the vicinity of the platform is densified, as shown in Figure 3 and Figure 4.

#### 2.3.3. Loads and Boundary Conditions

The front, top, and left and right sides of the computational domain are set as velocity inlets, with an incoming velocity *v* of 3 kn in the positive X-axis direction. The back end of the computational domain is set as a pressure outlet with a pressure of 0. The lower-end boundary of the computational domain simulates the seabed boundary and is set as a wall. The surface of the underwater bottom platform is set as a wall, and for viscous flows, the wall boundary is implicitly defined as a no-slip condition [8].

### 2.4. Structural Simulation Model

#### 2.4.1. Mesh Model

**Figure 5:**
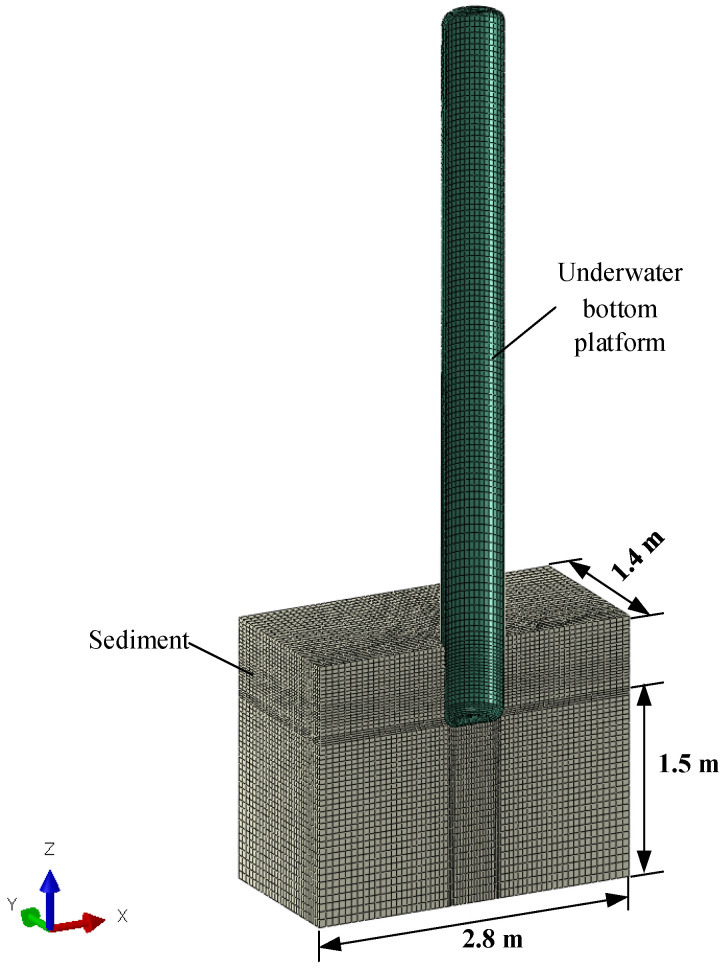
Schematic diagram of the mesh of the proposed structural simulation model.

Due to the symmetrical geometry, loads, and boundary conditions, only half of the model is used for modeling [9]. The underwater bottom platform is simplified as a thin-walled cylinder, and since its stiffness is much greater than that of the sediments, it is treated as a rigid body using a quadrilateral shell mesh, and the sediments are represented using pure hexahedral mesh. The entire mesh model is shown in Figure 5.

#### 2.4.2. Loads and Boundary Conditions

Before structural simulation calculations are performed, adjustments are made to the material density and additional concentrated mass of the underwater bottom platform to match its mass with a negative buoyancy of 450 kg and a center of mass displacement of 800 mm. The influence of buoyancy is not considered during calculations, and the negative buoyancy is effectively replaced by the gravity of the 450 kg mass of the platform, resulting in loads on the structure being the force exerted by the ocean current and gravity. The force exerted by the ocean current on the platform and its location are obtained from fluid calculation results.

**Figure 6:**
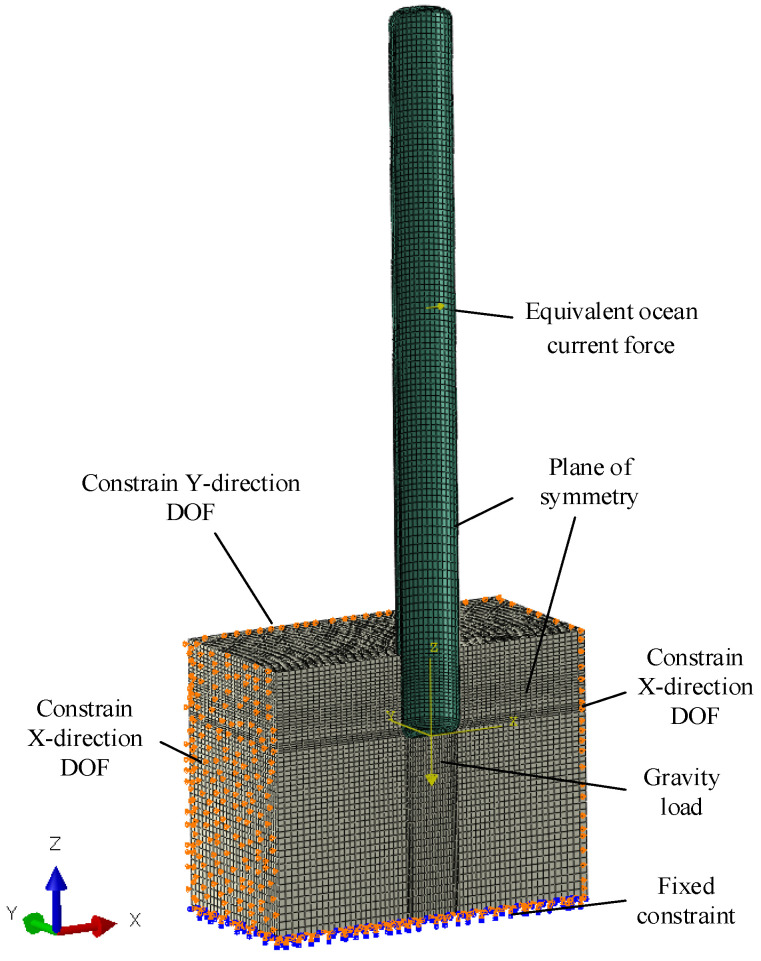
Schematic diagram of the load and boundary of the structural simulation model.

Boundary constraints and load application for the simulation model are shown in Figure 6. Symmetric constraints are applied to XZ-plane nodes on symmetric faces [10], free degrees of freedom are applied to nodes on both ends of the sediments in the X-direction, free degrees of freedom are applied to nodes on the right side of the sediments in the Y-direction, and fixed constraints are applied to nodes at the bottom of the sediments. Contact is defined between the sediments and the underwater bottom platform at their interface with a friction coefficient of 0.3.

#### 2.4.3. Sediment Constitutive Models and Parameters

The Mohr–Coulomb yield criterion is the most commonly used criterion for describing geotechnical engineering materials. The governing equation of this yield criterion [8] is

(2) $$f=\left(\sigma_{1},\sigma_{2},\sigma_{3}\right)=\frac{1}{2}\left(\sigma_{1}-\sigma_{3}\right)+\frac{1}{2}\left(\sigma_{1}+\sigma_{3}\right)\sin\phi -c\cos\phi$$

where $\sigma_{1},\;\sigma_{2},$ and $\sigma_{3}$ represent the first, second, and third principal stresses, respectively, and c and ϕ represent the cohesion and the friction angle, respectively.

**Figure 7:**
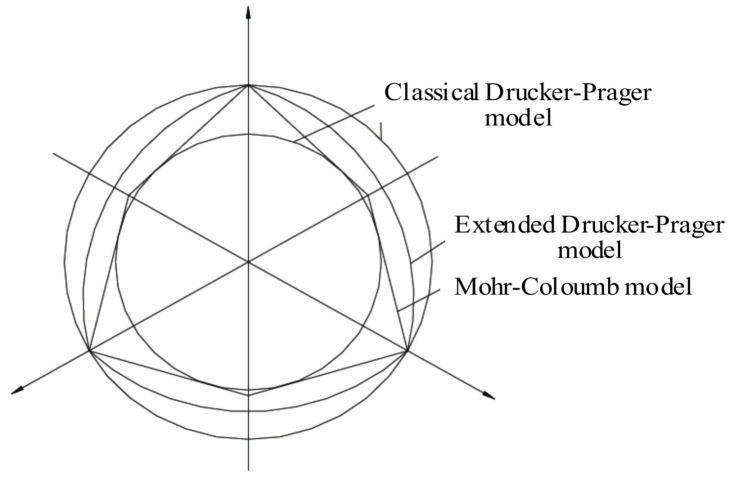
The yield planes corresponding to different yield criteria on the offset plane.

Since the Mohr–Coulomb yield surface has a hexagonal shape in flattened planes when it intersects with a circle in flattened planes, convergence difficulties can arise due to plastic flow directions at corners during plastic analysis. Based on this model, several modified models have been proposed [11], with the most typical being the Drucker–Prager model. The Drucker–Prager model defines a yield surface that circumscribes or inscribes Mohr’s hexagon on flattened planes through control equations [8] as follows:

(3) $$f=D_{1}I_{1}+\sqrt{J_{2}}-D_{2}=0$$

(4) $$I_{1}=\sigma_{1}+\sigma_{2}+\sigma_{3}$$

(5) $$J_{2}=\frac{1}{6}\left[{\left(\sigma_{1}-\sigma_{2}\right)}^{2}+{\left(\sigma_{2}-\sigma_{3}\right)}^{2}+{\left(\sigma_{1}-\sigma_{3}\right)}^{2}\right]$$

(6) $$D_{1}=\frac{2\sin\phi }{\sqrt{3}\left(3-\sin\phi \right)}$$

(7) $$D_{2}=\frac{6\cos\phi }{\sqrt{3}\left(3-\sin\phi \right)}$$

where $I_{1}$ is the first invariant of the stress tensor, and $J_{2}$ is the second invariant of the stress deviator. ABAQUS software 6.14 has extended and modified the classic Drucker–Prager model [12]. There are three forms on the meridian plane: straight line, parabola, and exponential. On the flattened plane, they are typically piecewise smooth curves with smooth connections between them. The three yield criteria on the flattened plane correspond to the yield lines shown in Figure 7.

When the shape of the yield surface of the extended Drucker–Prager model on the meridian plane is a straight line, its yield surface expression is

(8) F=t−ptanβ−c=0

(9) $$t=\frac{q}{2}\left[1+\frac{1}{k}-\left(1-k\right){\left(\frac{r}{q}\right)}^{3}\right]$$

In this formula, *q* represents the deviatoric stress; *k* is the ratio of the flow stress, which is the ratio of the three-axis tensile strength to the three-axis compressive strength, reflecting the impact of intermediate principal stress on yield; *r* is the third deviatoric stress invariant; *p* is the mean stress; *β* is the slope of the yield surface in the p–t stress plane, usually referring to the internal friction angle of the material; and *c* is the cohesion of the material.

The plastic potential surface expression of the linear Drucker–Prager criterion is

(10) G=t−ptanφ

In this formula, *φ* represents the dilatation angle.

## 3. Analysis of Simulation Results

The example adopts the cylindrical platform as the base, with a length of 6000 mm, a diameter of 534 mm, a negative buoyancy of 450 kg, and a center of mass distance of 800 mm. The burial depth of the base platform *d* is set to 0.5 m, 0.6 m, 0.7 m, and 0.8 m, respectively. The mud and sand adopt the linear Drucker–Prager constitutive model [13,14], and its material parameters are shown in Table 1.

### 3.1. Simulation Process

**Figure 8:**
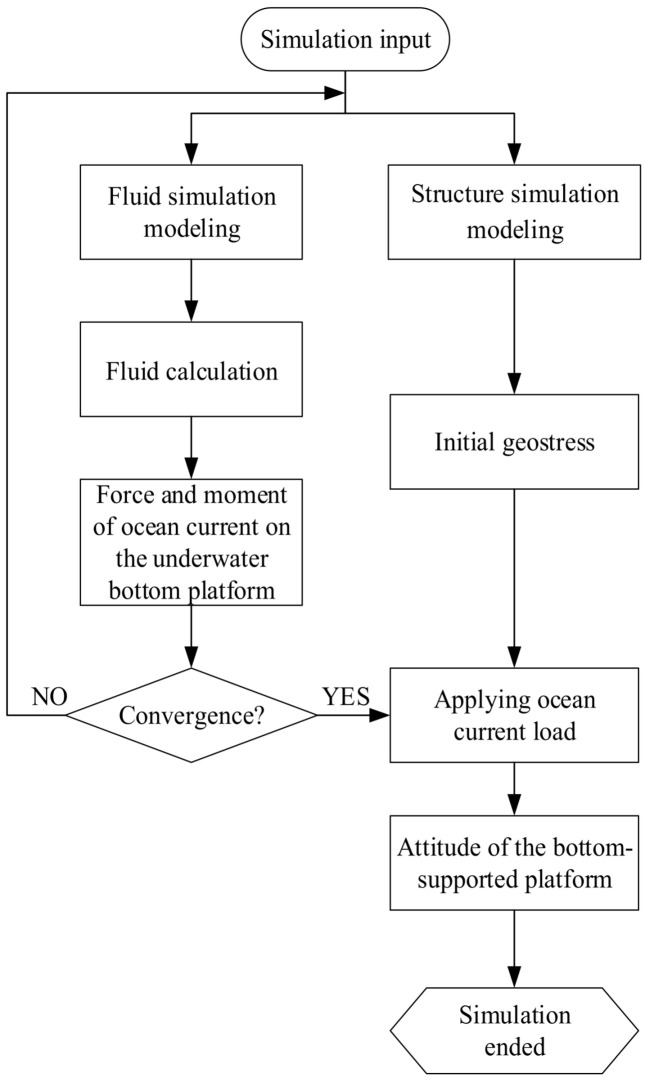
Schematic diagram of the proposed simulation calculation process.

The fluid simulation modeling of the underwater bottom platform is conducted using the CFD software STAR-CCM+ 11.04 [13] to calculate the force and torque acting on the platform under the action of a 3 kn current. Secondly, based on the FEM software ABAQUS 6.14, the equilibrium state of the platform and sediment under negative buoyancy and gravity is calculated (initial ground stress). Finally, the results of fluid simulation are used to apply the current load to calculate the posture of the platform. The simulation process for each burial depth of the submersible platform is shown in Figure 8.

### 3.2. Fluid Simulation Analysis

#### 3.2.1. Outer Flow Velocity

**Figure 9:**
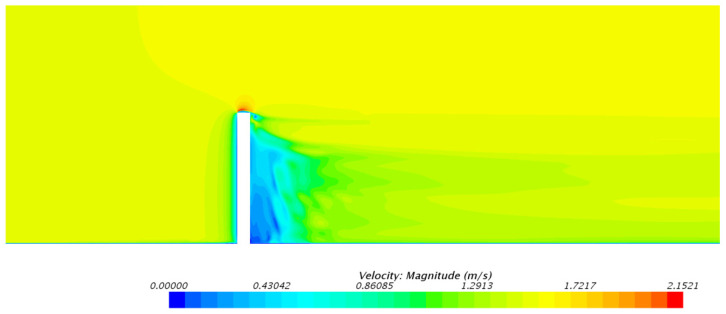
Velocity distribution on the mid-longitudinal plane of the external flow field around the underwater bottom platform.

**Figure 10:**
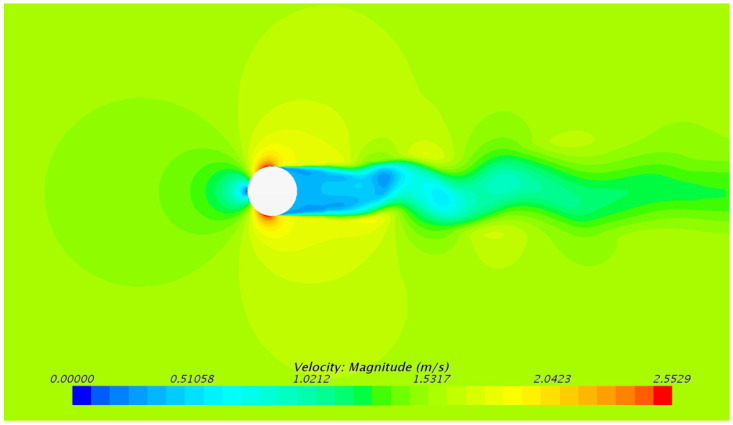
Velocity distribution on the horizontal plane (3 m from the seabed) of the external flow field around the underwater bottom platform.

The velocity distribution of the outer flow field of the underwater bottom platform at a burial depth of 0.5 m is shown in Figure 9 and Figure 10, and the velocity distribution of the outer flow field of the underwater bottom platform at other burial depths is similar. It can be seen that under the action of a uniform 3 kn current, the fluid velocity is larger on the left and right sides and on the top of the platform, while the fluid velocity is smaller on the upstream and downstream sides, and there is a distinct wake region on the downstream side.

#### 3.2.2. Surface Pressure on the Underwater Bottom Platform

**Figure 11:**
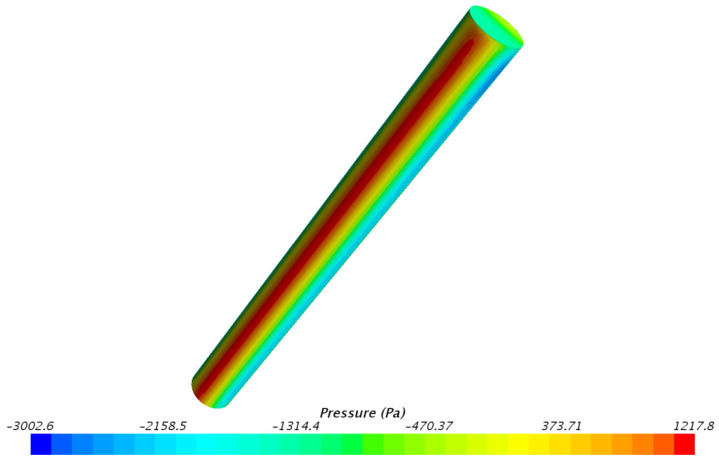
Pressure distribution on the surface of the underwater bottom platform.

When the burial depth is 0.5 m, the pressure contour plot on the outer surface of the underwater bottom platform is shown in Figure 11. The pressure distribution on the outer surface of the platform at other burial depths is similar [15]. It can be seen that under the impact of the current, the pressure on the central position of the upstream side of the platform is the largest at 1217.8 Pa.

Based on the pressure distribution on the underwater bottom platform, the equivalent force and torque can be calculated [16,17]. Under the action of a 3 kn current, the equivalent force and torque on the platform at different burial depths are shown in Table 2.

### 3.3. Analysis of Initial Equilibrium State

#### 3.3.1. Displacement of the Underwater Bottom Platform

**Figure 12:**
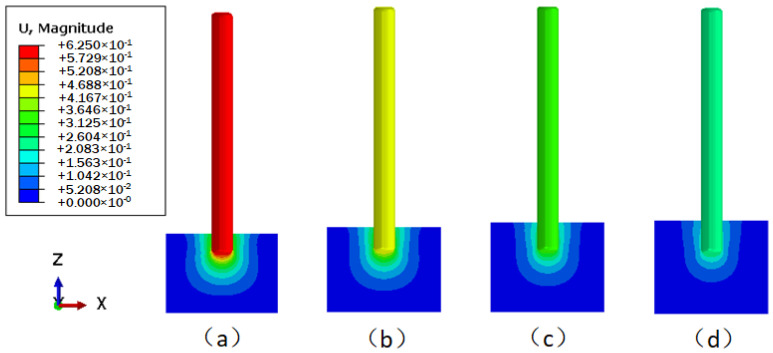
Displacement contours when the interaction between the underwater bottom platform and the sediment reaches equilibrium (mm): (**a**) buried 0.5 m; (**b**) buried 0.6 m; (**c**) buried 0.7 m; (**d**) buried 0.8 m.

**Figure 13:**
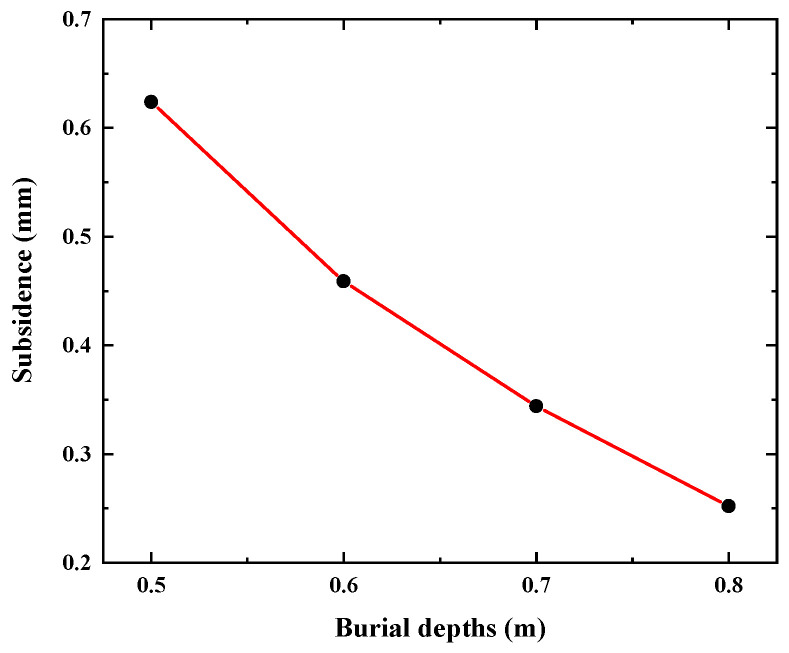
Subsidence of the underwater bottom platform under different burial depths.

The displacement contours of the underwater bottom platform achieving balance with the sediments at different burial depths are shown in Figure 12. It can be seen that after the platform is buried, it will continue to sink deeper into the sediments under the action of negative buoyancy, and the deeper the burial depth, the smaller the sinking amount. The change in sinking amount with burial depth is shown in Figure 13. When the burial depths are 0.5 m, 0.6 m, 0.7 m, and 0.8 m, the sinking amounts of the platform are 0.624 mm, 0.459 mm, 0.344 mm, and 0.252 mm, respectively.

#### 3.3.2. Sediment Stress

**Figure 14:**
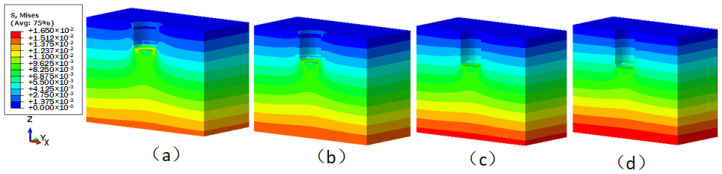
Stress distribution of the sediment when the interaction between the underwater bottom platform and the sediment reaches equilibrium (MPa): (**a**) buried 0.5 m; (**b**) buried 0.6 m; (**c**) buried 0.7 m; (**d**) buried 0.8 m.

The stress contours of the underwater bottom platform achieving balance with the sediments at different burial depths are shown in Figure 14. It can be seen that when the burial depth is less than 0.8 m, the stress at the contact area between the bottom surface of the platform and the sediments is greater than that at other locations at the same depth. However, when the burial depth is 0.8 m, the stress at the contact area is approximately equal to that at other locations at the same depth. This is because the surface sediments have a smaller self-weight and a looser structure, which is not sufficient to support the platform, so the surface sediments move downward and further compress with the underlying sediments. The deeper sediments have a greater self-weight and a denser structure, which is sufficient to support the platform without much downward movement. When the burial depths are 0.5 m, 0.6 m, 0.7 m, and 0.8 m, the maximum stresses of the sediments are 14.4 kPa, 15.1 kPa, 15.7 kPa, and 16.5 kPa, respectively, which are all below the yield stress of the sediments (26.1 kPa).

### 3.4. Analysis of the Ocean Current Effect

#### 3.4.1. Displacement of the Underwater Bottom Platform

**Figure 15:**
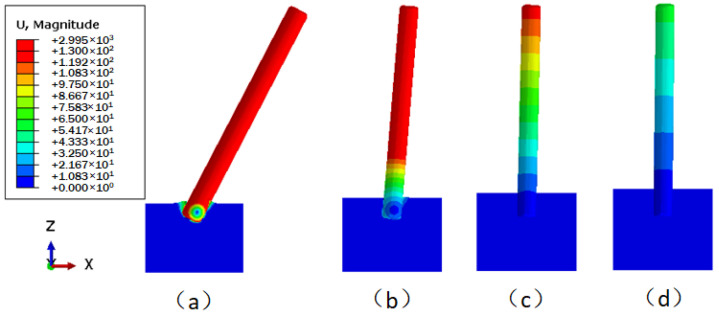
Displacement distribution of the underwater bottom platform under the effect of ocean current (mm): (**a**) buried 0.5 m; (**b**) buried 0.6 m; (**c**) buried 0.7 m; (**d**) buried 0.8 m.

**Figure 16:**
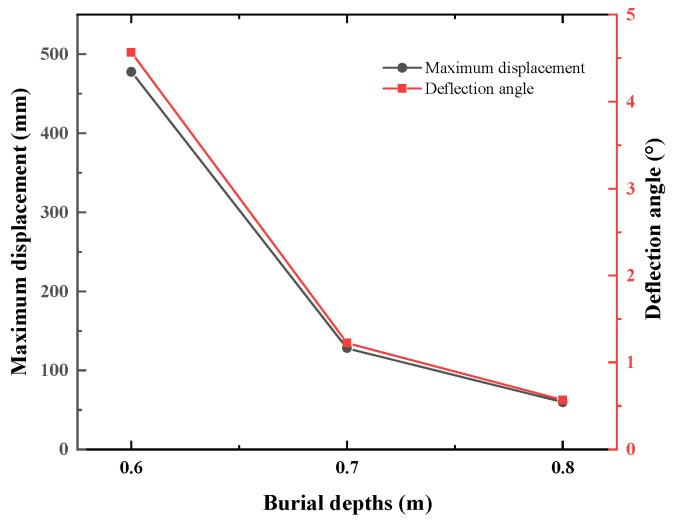
Maximum displacement and deflection angle of the underwater bottom platform with different burial depths under the effect of ocean current.

The displacement cloud images of the underwater bottom platform with different burial depths under a 3 kn sea current effect are shown in Figure 15. Figure 15a cannot converge during the calculation, indicating that when the burial depth is 0.5 m, the platform cannot be stabilized under the action of a 3 kn sea current. As can be seen from Figure 15b–d, the deeper the burial depth, the smaller the deflection of the platform. The maximum displacement and deflection angle vary with the burial depth, as shown in Figure 16. When the burial depths are 0.6 m, 0.7 m, and 0.8 m, the maximum displacements of the platform are 477.7 mm, 128.2 mm, and 59.6 mm, respectively, and the corresponding deflection angles are 4.567°, 1.224°, and 0.569°. To satisfy the stability requirement of the posture of the platform under a 3 kn sea current, the deflection angle should be less than 5°; thus, the burial depth of the platform should be greater than 0.6 m.

#### 3.4.2. Sediment Stress and Strain

**Figure 17:**
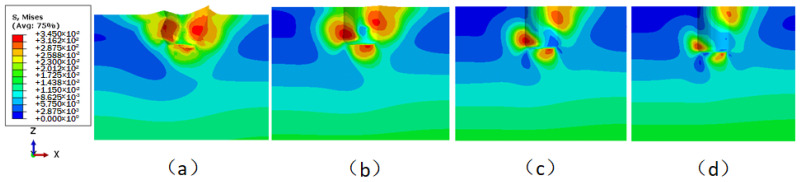
Stress distribution of the sediment under the effect of ocean current (MPa): (**a**) buried 0.5 m; (**b**) buried 0.6 m; (**c**) buried 0.7 m; (**d**) buried 0.8 m.

The stress cloud images of the sediment with different burial depths under a 3 kn sea current effect are shown in Figure 17. Figure 17a cannot converge during the calculation, indicating that when the burial depth is 0.5 m, the underwater bottom platform cannot be stabilized under the action of a 3 kn sea current [18,19,20]. Visible locations with larger sediment stress are located below the upstream side, the bottom rear, and the upstream side of the backflow of the platform. These are the main contact areas between the platform and the sediment after the deflection, and as the burial depth increases, the larger stress area becomes more concentrated. When the burial depths are 0.5 m, 0.6 m, 0.7 m, and 0.8 m, the maximum stresses of the sediment are 33.2 kPa, 34.5 kPa, 33.2 kPa, and 31.3 kPa, respectively, all exceeding the yield stress (26.1 kPa), resulting in plastic deformation.

**Figure 18:**
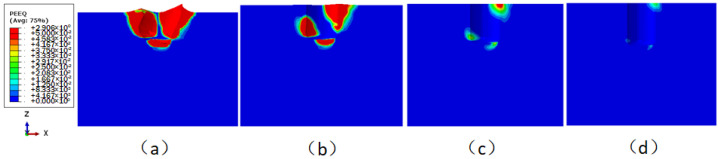
Equivalent plastic strain distribution of the sediment under the effect of ocean current: (**a**) buried 0.5 m; (**b**) buried 0.6 m; (**c**) buried 0.7 m; (**d**) buried 0.8 m.

The equivalent plastic strain cloud images of the sediment with different burial depths under a 3 kn sea current effect are shown in Figure 18. The equivalent plastic strain is used to measure the amount of plastic deformation of materials [21], and it is a cumulative quantity. Visible locations with plastic deformation are located in the main contact areas between the underwater bottom platform and the sediment after deflection. The maximum equivalent plastic strains of the sediment are 2.91, 0.28, 0.02, and 0.02, respectively.

## 4. Conclusions

This paper takes a cylindrical underwater bottom platform as the research object. Through fluid simulation, it investigates the velocity distribution and force acting on the platform at different burial depths of 0.5 m, 0.6 m, 0.7 m, and 0.8 m under a 3 kn sea current. Through structural simulation, it studies not only the initial equilibrium state and the deflection angle of the buried platform but also the sediment stress and strain under the ocean current.

The innovation of this paper can be summarized as follows: The Drucker–Prager constitutive model is used to innovatively describe the plastic mechanical characteristics of seabed sediment. The simulation evaluates the equivalent plastic strain of sediment at different burial depths of the underwater bottom platform under the combined action of ground stress and ocean current, providing technical support for the application of underwater bottom platforms as engineering sensors.

The following conclusions can be obtained:

(1) Under the action of a 3 kn sea current, the fluid velocity on the upstream and downstream sides of the platform is relatively small, while the fluid velocity on the left and right sides and at the top is relatively large. There is a clear wake zone formed on the downstream side. The pressure on the central position of the upstream side of the platform is the largest under sea current impact.
(2) After burial, the platform will continue to sink under negative buoyancy until the negative buoyancy and sediment force reach equilibrium. The deeper the burial depth of the platform, the smaller the sinking amount. Under the burial depths of 0.5 m–0.8 m, the equilibrium sediment stress does not reach the yield stress.
(3) The deeper the burial depth of the platform, the smaller the deflection caused by the 3 kn sea current. When the burial depth is greater than 0.6 m, the deflection angle of the platform is less than 5°, maintaining a stable posture.
(4) Under the action of a 3 kn sea current, the platform deflects, and the locations with larger sediment stress are located below the upstream side, at the rear of the bottom side, and above the downstream side of the platform. The deeper the burial depth, the more concentrated the region with larger stress. Under the burial depths of 0.5 m–0.8 m, the sediment stress reaches the yield stress, resulting in plastic deformation.

## Figures and Tables

**Table 1:** The sediment material parameters.

Density (g/cm^3^)	Elastic Modulus E (MPa)	Poisson’s Ratio	Cohesion C (MPa)	Internal Friction Angle β (°)	Flow Stress Ratio (k)	Expansion Angle φ (°)	Compressive Yield Stress (MPa)
1.8	3.98	0.4	0.011	9.6	0.778	9.6	0.0261

**Table 2:** The sediment material parameters.

Burial depths (m)	0.5	0.6	0.7	0.8
Equivalent force (N)	1450	1380	1350	1338
Equivalent moment of force (Nm)	4060	3920	3835	3660

## Data Availability

Data are available upon request from the authors.
